# Meditation experts try Virtual Reality Mindfulness: A pilot study evaluation of the feasibility and acceptability of Virtual Reality to facilitate mindfulness practice in people attending a Mindfulness conference.

**DOI:** 10.1371/journal.pone.0187777

**Published:** 2017-11-22

**Authors:** María V. Navarro-Haro, Yolanda López-del-Hoyo, Daniel Campos, Marsha M. Linehan, Hunter G. Hoffman, Azucena García-Palacios, Marta Modrego-Alarcón, Luis Borao, Javier García-Campayo

**Affiliations:** 1 Hospital General de Catalunya, Barcelona, Spain; 2 Universidad de Zaragoza, Zaragoza, Spain; 3 Red de Investigación en Atención Primaria (REDIAPP), Zaragoza, Spain; 4 Universitat Jaume I, Castellón, Spain; 5 Behavioral Research & Therapy Clinics, University of Washington, Seattle, WA, United States of America; 6 Virtual Reality Research Center at the Human Photonics Lab, Mechanical Engineering, University of Washington Seattle, Seattle, WA, United States of America; 7 CIBER of Physiopathology of Obesity and Nutrition CIBERobn, CB06/03 Instituto de Salud Carlos III, Madrid, Spain; 8 ISS Aragón. Hospital Universitario Miguel Servet, Zaragoza, Spain; Waseda University, JAPAN

## Abstract

Regular mindfulness practice benefits people both mentally and physically, but many populations who could benefit do not practice mindfulness. Virtual Reality (VR) is a new technology that helps capture participants’ attention and gives users the illusion of “being there” in the 3D computer generated environment, facilitating sense of presence. By limiting distractions from the real world, increasing sense of presence and giving people an interesting place to go to practice mindfulness, Virtual Reality may facilitate mindfulness practice. Traditional Dialectical Behavioral Therapy (DBT®) mindfulness skills training was specifically designed for clinical treatment of people who have trouble focusing attention, however severe patients often show difficulties or lack of motivation to practice mindfulness during the training. The present pilot study explored whether a sample of mindfulness experts would find useful and recommend a new VR Dialectical Behavioral Therapy (DBT®) mindfulness skills training technique and whether they would show any benefit. Forty four participants attending a mindfulness conference put on an Oculus Rift DK2 Virtual Reality helmet and floated down a calm 3D computer generated virtual river while listening to digitized DBT® mindfulness skills training instructions. On subjective questionnaires completed by the participants before and after the VR DBT® mindfulness skills training session, participants reported increases/improvements in state of mindfulness, and reductions in negative emotional states. After VR, participants reported significantly less sadness, anger, and anxiety, and reported being significantly more relaxed. Participants reported a moderate to strong illusion of going inside the 3D computer generated world (i.e., moderate to high “presence” in VR) and showed high acceptance of VR as a technique to practice mindfulness. These results show encouraging preliminary evidence of the feasibility and acceptability of using VR to practice mindfulness based on clinical expert feedback. VR is a technology with potential to increase computerized dissemination of DBT® skills training modules. Future research is warranted.

## Introduction

Mindfulness has been defined as “the act of consciously focusing the mind in the present moment without judgment and without attachment to the moment” [1]. Mindfulness refers to the self-regulation of attention to one's experiences in the present moment with curiosity, openness and acceptance [2]. Mindfulness-based interventions, such as Mindfulness-Based Stress Reduction [3] are used by the general population to increase the health of non-patients via “wellness” programs [4]. Research in non-patient populations indicates that mindfulness practice helps decrease depressive symptoms and rumination [5] as well as increase positive affect [6, 7] in non-clinical populations. Mindfulness practice also leads to multiple health benefits: increasing immune functioning [8]; improving well-being [9, 10] and increasing working memory and sustained attention [5, 7]. A review study indicates that participation in a Mindfulness Based Stress Reduction program yields benefits for non-patients, such as decreasing stress and burnout of health care practitioners [11, 12]. Similar benefits of mindfulness have been found across different cultures [13].

Despite its benefits, people being trained to practice mindfulness often report that it is very difficult to sit still, be fully present, and focus their attention on their own breathing or other mindfulness exercises. DBT® mindfulness skills training was developed to help train mindfulness skills to people who have difficulty concentrating [1].

In clinical studies, traditional DBT® skills training is proving to be a valuable element of DBT® for treating borderline personality disorder [1, 14, 15], and a growing number of other psychological problems [16]. DBT mindfulness skills training sessions are brief (e.g., 10 minute mindfulness sessions) and easy to apply (specific exercises with instructions) because they were designed for people who show difficulties practicing mindfulness. Traditional training in DBT mindfulness skills training has shown health benefits such as increased attention, as well as reduced impulsivity for clinical population characterized by emotion dysregulation [17–19]. Although DBT® mindfulness skills training sessions are brief and easy to teach, populations with severe psychological problems who need mindfulness the most often still have trouble concentrating, and/or find mindfulness difficult or boring. As a result, many people who could benefit greatly from mindfulness training either cannot do mindfulness, or they quit the training prematurely.

During traditional DBT® mindfulness skills training sessions with no VR, participants keep their eyes open during mindfulness. They often simply sit in a chair in a comfortable posture, sitting up with their back reasonably straight, with their feet on the floor, and their hands clasped together. As they practice mindfulness, they may be directed by the instructor to focus their attention on their breathing rate (or some other object of focus). Although the instructor attempts to provide a quiet environment, unwanted sights and sounds often intrude, and can distract the patient’s attention away from their focus (e.g., their focus on breathing). One of the main goals of the mindfulness training sessions is to train people how to bring their attention back to the object of focus, when their mind wanders. For example, participants may hear someone’s cell phone ring, and while the phone is ringing, the trainee may be less able to focus on their own breathing. But that’s ok, the trainee is instructed to simply and gently bring their attention back to their breathing, if their attention wanders.

When people practice DBT mindfulness in a classroom, living room, or therapists office, they are typically encouraged to focus their gaze in one direction (e.g., one leg of the chair in front of them), and sit in once place. The amount of sensory information coming into the patient's brain is lower than usual. Although controlled breathing is therapeutic, breathing patterns are not the most interesting focal objects, especially for novice inexperienced participants. Although the goal of mindfulness may be to remain fully alert and “present” in the current moment, the traditional practice of mindfulness may leave novice trainees less alert than usual, in a state of temporary dormancy, due to the relative lack of sensory input during DBT® mindfulness skills training sessions. The lack of sensory input may be one reason that unhelpful internal thoughts (e.g. images of a previous traumatic or distressful event) are more likely to surface to consciousness during mindfulness. Eventually, the therapist will help the patient deal with these difficult thoughts and emotions (e.g., during standard DBT), but the initial focus during DBT® mindfulness skills training module is on increasing sustained attention, with acceptance.

**Fig 1:**
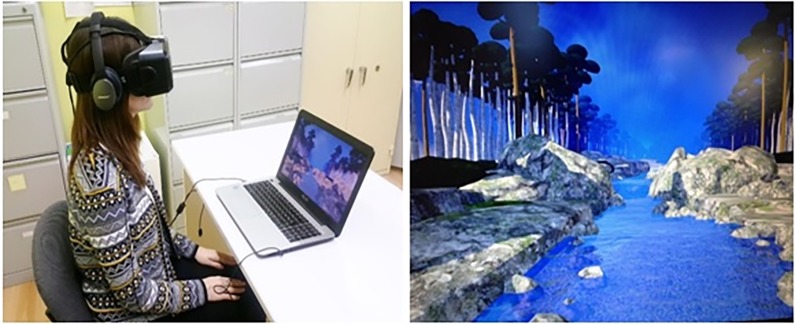
Image on left. 3rd International Mindfulness Congress' participant testing Virtual Reality DBT mindfulness skills training. Image on right. A screenshot of the Virtual Reality world the participant watched through Oculus Rift DK2 VR goggles while listening to Dr. Linehan's DBT® Mindfulness Skills™ audios (http://behavioraltech.org). Image by www.bigenvironments.com, copyright Hunter Hoffman, www.vrpain.com. Note: The individual in this manuscript has given written informed consent (as outlined in PLOS consent form) to publish these case details.

The theoretical rationale behind why Virtual Reality might enhance Dialectical Behavioral Therapy (i.e., DBT®) mindfulness skills training is as follows. In contrast to the traditional mindfulness with no VR described above, during our new VR DBT® mindfulness skills training, trainees practice mindfulness while in virtual reality. Immersive virtual reality is a paradigm shift in how people interact with computers. Immersive virtual reality is designed to give the computer user a *sense of presence*, the illusion of going inside the computer generated world, as if it is a place they are visiting [20]. Virtual Reality is a new very attention grabbing technology. People put on VR goggles (see Fig 1).

Participants look into the VR goggles, and they see computer-generated images. What they see in VR changes depending on which direction they are looking. The virtual world hardware and software are designed to give the participant the illusion that they are “there” in the computer generated world. Using head tracking technology, participants view of the virtual world changes in real time, as they move their head orientation/look around. For example, the person starts out looking straight down a river in virtual reality. They can see trees on the sides of the river, ripples in the surface of the water; they can see rocks in the bottom of the river. Initially, this may seem like they are watching a movie and are about to float down the river in a movie. But VR is more immersive than a movie. Unlike watching a movie, when someone moves their head in virtual reality, what they see changes accordingly, as if they were really there in the computer generated world. If the trainee rotates their head orientation to the left, now they can see virtual mountains that were not visible when they were looking straight down river, if they look down, they see rocks in the bottom of the river. If they lean forward as they are looking down, the river seems to get closer.

Users practice mindfulness while “being there” in a computer generated virtual environment. Their visual cortex is stimulated by the visual input they see in the VR helmet, their auditory cortex is stimulated by sounds they hear (e.g., sounds of nature, sounds of water, and birds chirping in VR). Virtual Reality uses up a lot of attentional resources [21], making it less likely that attention will drift to sights and sounds in the real world, and the increased sensory input may reduce distressing thoughts or emotions, while in VR. For example, VR is so attention grabbing that it is being used to distract severe burn patients from their pain during severe burn wound cleaning, and burn patients report large reductions in the amount of time they spend thinking about their pain during painful medical procedures [22–25]. However, although VR helps user paying attention to the experience, the goal of VR mindfulness skills is not to distract. Random instructions to redirect attention to specific stimuli (e.g. sounds, visuals) while in the virtual experience were developed to target active control of attention. In summary, the logic behind of using VR to practice DBT mindfulness is that the person’s mind is less likely to wander, and they are more likely to be able to “be in the present moment” if they are focusing their attention on something in a virtual world specifically designed for eliciting mindfulness.

The main objective of the present study was to explore the feasibility, acceptability and the potential benefits of using VR to facilitate DBT® mindfulness skills training in a non-clinical sample of people who already practice mindfulness. We predicted that: 1) Participants would accept VR for learning DBT® mindfulness skills; 2) DBT® mindfulness skills training facilitated with virtual reality would decrease negative emotions (e.g., anger) after each session; 3) VR DBT® mindfulness skills training would increase the participants' self-reported state of mindfulness and sense of presence and would increase positive emotions after each session.

## Materials and methods

### Participants

Participants were 44 attendees of the 3rd International Meeting on Mindfulness held in Zaragoza, Spain, in June 2016. Mean age was 45.32 (SD = 13.20) ranging from 21 to 69. There were 16 men (36.4%), and 28 women (63.6%). Most of the participants were married (N = 29; 65.9%), 11 single (25%), and 4 participants were separated (9.1%). The average education level was a bachelor degree. The sample's employment status was: Students (7.3%), unemployed (9.8%), employed (80.5%), or retired (2.4%).

### Design

We conducted a pilot/feasibility study as a first step toward later conducting a full-scale trial [26] with clinical populations. Using a within-subjects design, each participant filled out ratings before and after a 10 minute VR DBT® mindfulness skills training session.

### Study procedures

#### Recruitment of participants

One week before entering into the study, participants were invited via email to participate in the study. In the email, participants were informed about the general aim of the study and were asked to complete a survey regarding their previous meditation/mindfulness experience and demographic information. They were also required to choose a date and time (from a wide list of options) during the 3rd International Meeting on Mindfulness, to test the VR mindfulness intervention. As an incentive, participants were offered the opportunity to take part in a raffle of a set of mindfulness books valued at 200 Euros.

#### Procedure

Participants gave written informed consent prior to inclusion in this study. The Ethical Review Board of the regional health authority (Zaragoza, Spain) approved the study. All clinical investigation were conducted according to the principles expressed in the Declaration of Helsinki.

Subjects participated in the VR mindfulness intervention during one of the three days of the congress. Each experimental test was supervised by a research assistant collaborating in the study. The intervention for each participant lasted around 25 minutes: 15 minutes to orient participants about the study and to complete the brief pre- and post-VR assessments, and 10 minutes to go through the VR mindfulness intervention. Participants completed a brief battery of measures (described in the assessment section below) before and after receiving the VR mindfulness intervention. Several measures were shortened to reduce the burden on participants.

#### VR DBT mindfulness intervention

As shown in Fig 1, each participant sat in a chair and wore a pair of wide field of view (80 diagonal FOV per eye) Oculus Rift DK2 VR goggles with head mounted display, with head tracking that allowed them to see the 3D computer generated river DBT® VR World with the help of a MSI GT Series GT72 Dominator Pro G-1252 Gaming Laptop 6th Generation Intel Core i7 6700HQ (2.60 GHz) 16 GB Memory 1 TB HDD 512 GB SSD NVIDIA GeForce GTX 980M 4 GB GDDR5 17.3" Windows 10 Home 64-Bit while listening to one of the DBT® mindfulness skills practices using Bose Q25 headphones. The visuals of (see Fig 1) were designed to give the patient the illusion of going inside the 3D computer generated world, where they floated slowly down an animated computer generated river (with ripples) in VR, with trees, boulders, and mountains (VR World/visuals created and copyrighted by BigEnvironments.com using Unity3D software, see also vrpain.com).

While wearing the VR goggles and while floating down the river in virtual reality [27], participants listened to DBT® mindfulness skills training modules (copyrighted by Marsha Linehan). The audios used during VR DBT® mindfulness skills training, were a Spanish adaptation and translation of the three DBT® mindfulness skills training audios. The visuals and the audios were mixed together and were presented as a multisensory experience by the VR software program (built using Unity3D world building software). In a previous case study [27], a patient experienced all three worlds for training DBT skills, on separate weekly visits. In the current study, due to time constraints and scheduling restrictions, there was not time for each participant to experience each of the VR DBT Skills worlds. Participants (conference attendees) listened to one of the three 10 minute DBT® mindfulness skills training audios (randomly assigned by a research assistant): Wise Mind, Observing Sound, or Observing visuals, while being immersed in a Virtual Reality system (pooled for analyses). Wise mind is the “synthesis or integration of opposites: emotion mind and reasonable mind” [1]. The *wise mind* audio, is based on the DBT® mindfulness skills training exercise called "Stone flake on the lake" [1] but adapted to match the visuals of floating down the river. In other words, the VR computer controlled digitized audio was slightly adjusted to be consistent with what people saw in VR (as their viewpoint floated down the river in the virtual reality goggles).

The *Observing sound*s audio consisted of noticing sounds, and repeatedly bringing attention back to sounds every time the mind wanders off [28]. For the VR *Observing sounds*, the participant stayed at the top of the river during the instruction phase. At the end of the instruction phase, the ancient bell rang three times, and as instructed, the trainee began slowly descending down the river in virtual reality. At the same time they began descending, the participant began to hear digital sounds from nature that were customized to match the visuals in VR: sounds of water flowing, birds chirping, and crickets, as they descended down the river. While listening to these sounds of nature in their VR earphones, they could see visuals of a river and trees, mountains in the background, consistent with the sound effects. The result was a subtle multisensory input from visual and digital auditory stimuli all presented to the participant in the VR helmet and Bose Q25 earphones via from the virtual reality software. Instructions to develop active attention to the sounds while descending to the virtual world were added (e.g. "Focus on the sounds, just the sounds…").

The goal of the *observing visuals* audio was for the person to just observe what he/she saw, to notice, not to let their attention get fixed upon anything, and to bring their attention back, if it became distracted. The “Observing visuals” script was written by Marsha Linehan in her office as she experienced the VR Riverin the VR goggles. Participants were instructed to “pay attention to what you see, as you slowly float down the river. Focus on the sun, the intensity of the colors, the textures of what is going by, see the sky, the clouds, the colors….if your attention wanders, bring it back, gently….to the water.”

### Assessments

#### Demographic information and meditation frequency

The demographics scale gathered information about gender, age, marital status, educational level, employment status, type of work, type of working day and if they have any kind of chronic pathology. “Meditation frequency during the last year” was assessed using a brief scale adapted from [29]. Participants reported whether they typically meditated every day, 3 or 4 times a week, or less than 3 times per week during the last year.

#### Emotional state

Using a visual analog scale (VAS) [30] the emotional state scale was designed to evaluate the intensity of different emotions before and after the virtual reality intervention. In the current study, to reduce the time burden on the volunteer participants, a briefer (7 item emotion) version of the original measure (16 item emotion) was used. Participants were asked to rate on a 1–7 points Visual Analog Scale (1 = not feeling the emotion at all; 7 = feeling the emotion extremely), how they felt at that *moment* for the following emotions: happiness, sadness, anger, surprise, anxiety, relax/calm vigor/energy. This shorter 7-item scale has been successfully used in previous studies [31].

#### State of mindfulness

State of mindfulness was measured using an adaptation (shortened version) of the MAAS (Mindful Attention Awareness Scale) [32–34]. The MAAS is a 15-item scale designed to assess a core characteristic of mindfulness (i.e. “an open or receptive awareness”…”which may be reflected in a more regular or sustained consciousness of ongoing events and experiences” [32]. When collapsed across time, the internal consistency (alpha) of state mindfulness in the original measure, was .92 [32]. The five items (3, 8, 10, 13, and 14) designated from Brown & Ryan (2003) to assess *state mindfulness* were used in this study [32]. Each was rated on a 7-point scale anchored at 0 (*not at all*), 3 (*somewhat*), and 6 (*very much*). A state mindfulness score was derived by first reverse scoring some of the items, as appropriate, and then averaging responses; higher scores reflected more mindful states.

#### Sense of presence

The sense of presence questionnaire was composed of 3 items with a 7-point likert scale ranging from 1 to 7. The 3 items were adapted from the Slater, Usoh & Steed questionnaire [20], changing Slater et al.’s wording somewhat during the translation from English to Spanish. Participants were required to provide ratings on these three questions: 1.*“Rate your sense of being in the virtual environment*. *1 = “not at all”*, *7 = very much*.*”;* 2.*“To what extent were there times during the experience when the virtual environment was reality for you*?*” 1 = “at no time”*, *7 = “almost all of the time”;* 3.*“When you think back to the experience*, *do you think of the virtual environment more as images that you saw or more as some place that you visited*? 1 = something I saw, 7 = some place that I visited. This Spanish adaptation/version of this measure has been previously used to measure user's presence in virtual reality [31, 35].

#### Acceptability of the intervention

An adaption of the Credibility/Expectancy rating [36–37] was used. Participants completed this scale after the intervention. The adapted questionnaire was composed of 4 items rated from 0 (‘not at all’) to 10 (‘very much’). The items assessed the participant’s level of satisfaction with the intervention; how logical and how recommendable is the intervention; the extent to which the intervention is considered useful for practicing mindfulness; and the extent to which the participant thinks that the intervention was difficult to manage or aversive. The adaptation of these scales has been used in previous studies assessing virtual reality [37–38].

#### Experience on the use of technologies

A brief version of the Independent Television Company SOP Inventory, (ITC-SOPI) [39–40] was used to evaluate previous experience with technologies. We selected 4 questions to assess: 1) Experience with computers (answers choices were: none, basic, intermediate, and expert); 2) Knowledge about 3-D images (none, basic, intermediate, and expert); 3) Frequency of playing videogames a) never, b) occasionally (once or twice a month), c) frequently, but less than 50% of the days, d), 50% of the days or more); 4) Knowledge about VR (none, basic, intermediate, and expert). Preliminary analyses of previous studies demonstrated good reliability (Cronbach's alphas of the subscales ranged from 0.77 to 0.94) and validity for the ITC-SOPI [40].

### Statistical analyses

Descriptive statistics were calculated to explore demographic characteristics, sense of presence and acceptability of VR. Paired Student’s *t*-tests were used to assess the effects of VR mindfulness intervention *on state of mindfulness and emotional state* outcome measures. Effect sizes with Cohen’s *d* were reported [41–42]. In addition, Pearson correlations were conducted to explore relationships between changes in state of mindfulness and emotional state, the sense of presence and acceptability of VR system to practice mindfulness. Differences between pre- and post-intervention scores were calculated to estimate the change in state of mindfulness and emotional state. Total scores and items from the acceptability scale were included for correlation analyses. All analyses were conducted based on completers using IBM SPSS statistics 23 for Windows. Experimental data are available within the Supporting Information files (S1 File).

## Results

### Meditation data and experience using VR systems at baseline

Thirty four participants (77.3%) reported that they meditated daily or for at least three or four times per week, on average, during the last year; and 10 participants (22.7%) reported they meditated less than 3 times per week during the last year. As shown in Table 1, most participants reported an intermediate amount of experience using computers and basic (little) or no knowledge about VR and 3D images. Moreover, most participants did not usually play videogames.

**Table 1:** Experience using computers and VR systems.

*Items*	*Expert*	*Intermediate*	*Basic*	*None*
*Experience with computers*	18.6%	58.1%	23.3%	-
*Knowledge about 3-D images*	2.3%	4.7%	27.9%	65.1%
*Knowledge about VR*	4.7%	7%	27.9%	60.5%
*Frequency of playing videogames*	*50% of the days or more*	*Frequently*	*Occasionally*	*Never*
	-	4.7%	30.2%	65.1%

### Effects of VR to practice mindfulness

#### State of mindfulness

As presented in Table 2, participants reported significantly higher state mindfulness levels after the VR mindfulness intervention (t(42) = 4.431; *p* < .001; SE = .20) with medium to large effect size (*d* = .75).

**Table 2:** Means with standard deviation (SD) and within-group comparisons for *state of mindfulness* and *emotional state*.

	Pre		Post		Pre vs. Post			
	Mean	SD	Mean	SD	95% CI for Mean Difference	t	df	*d*
**State of Mindfulness**	4.10	1.12	4.97	1.01	-1.26, -.47	4.43***	42	0.76
**Emotional State**								
*Joy*	4.95	1.11	5.02	1.42	-.44, .30	.38	42	0.06
*Sadness*	1.83	1.17	1.30	.56	.19, .82	3.25**	42	0.44
*Anger*	1.40	.98	1.14	.35	.00, .50	2.04*	42	0.28
*Surprise*	2.86	1.54	3.23	1.96	-.86, .11	1.53	42	0.24
*Anxiety*	2.16	1.29	1.49	1.06	.19, 1.15	2.81**	42	0.51
*Relax*	4.67	1.32	5.58	1.34	-1.40, -.41	3.68**	42	0.68
*Vigor*	4.81	1.20	4.79	1.42	-.40, .45	.10	42	0.02

Note: M = mean; SD = Standard deviation; t(df) = t-value; *d* = Cohen’s d. CI = Confidence Interval.

* *p* < .05

**p< .01

*** *p* < .001.

#### Emotional state

Results revealed significant changes from pre to post VR mindfulness practice on *emotional state* outcomes (see Table 2 for details). Specifically, paired *t*-test comparisons showed that participants reported significantly lower *sadness* (t(42) = 3.250; *p* < .01; SE = .16; *d =* .44), *anger* (t(42) = 2.048; *p* < .05; SE = .13; *d =* 0.28), *anxiety* (t(42) = 2.818; *p* < .01; SE = .24; *d =* 0.51) and felt significantly more *relaxed/calm* (t(42) = 3.681; *p* < .01; SE = .25; *d =* 0.68) after the VR DBT® intervention.

#### Sense of presence

Results of descriptive statistics indicated medium to high scores for the *Sense of Presence* outcome followed VR mindfulness:

1. *Rate your sense of being in the virtual environment*. *1 = not at all; 7 = very much*. Mean = 5.5 (SD = 1.07).
2. *To what extent were there times during the experience when the virtual environment was reality for you*? *1 = at no time*, *7 = almost all of the time*. Mean = 4.33 (SD = 1.71).
3. *When you think back to the experience*, *do you think of the virtual environment more as images that you saw or more as some place that you visited*? *1 = something I saw; 7 = some place that I visited*. Mean = 4.12 (SD = 1.74).

### Acceptability of VR system to practice mindfulness

Results of descriptive statistics revealed high ratings from participants attending the mindfulness conference regarding the acceptability of VR mindfulness. Specifically, on a zero to 10 where zero = “not at all” and 10 = “very much:”, participants gave high ratings regarding *how logical the intervention seemed* (M = 7.31; SD = 2.10); *how confident they felt about the VR DBT® Mindfulness intervention* (M = 7.33; SD = 2.10); *whether they would recommend the VR system to others* (M = 7.38; SD = 2.52) and *VR’s usefulness for practicing mindfulness* (M = 7.33; SD = 2.14).

Results of correlations analysis between changes in the state of mindfulness and emotional state, sense of presence and acceptability of VR system to practice mindfulness are presented in Table 3.

**Table 3:** Correlations between changes in state of mindfulness and emotional state, the sense of presence and acceptability of VR system to practice mindfulness.

	*State of mindfulness*	*Acceptability*				
		Logic	*Confident*	*Recommend to others*	*VR’s usefulness*	*Total Score*
***State of Mindfulness***	–	.168	-.036	.098	.036	.074
**Emotional State**						
*Joy*	-.012	.306*	.391*	.142	.365*	.319*
*Sadness*	.088	-.013	.015	-.173	.037	-.043
*Anger*	.112	-.036	-.075	-.220	-.018	-.101
*Surprise*	-.118	.248	.297	.050	.357*	.250
*Anxiety*	-.231	-.200	-.224	-.277	-.184	-.243
*Relax*	.365*	.257	.336*	.317*	.309*	.332*
*Vigor*	.298	.169	.317*	.135	.320*	.251
**Sense of presence**						
*Q1*	.243	.463**	.512**	.537**	.568**	.566**
*Q2*	.203	.236	.138	.220	.210	.219
*Q3*	.168	.336*	.302	.295	.336*	.344*
*Total score*	.242	.399**	.352*	.393*	.417**	.424**

Note: VR = Virtual Reality. Q = question.

*p < .05

**p < .01.

## Discussion

The current pilot acceptability/feasibility study explored whether people who already practiced mindfulness on a regular basis were willing and able to try practicing Dialectical Behavioral Therapy mindfulness skills training while in virtual reality, and whether they reported any benefits afterwards.

With the current excitement and enthusiasm for VR in the clinical area, a lot of companies are developing VR applications that variously use a range of monikers such as “relaxation”, “mediation”, or “mindfulness” and are making claims about them without any data to support those claims. Thus, there is a need of conducting research studies to document or validate efficacy of these applications.

At the time of writing, this is the first expert user study indexed on PubMed to explore doing DBT mindfulness while in virtual reality. Our sample of 44 non-clinical participants attending a mindfulness conference accepted the use of VR as a new technique for practicing mindfulness. Participants were willing to try virtual reality DBT® mindfulness, supported VR’s usefulness for practicing mindfulness, and they highly recommended the VR system to others. Participants rated their illusion of going inside the computer generated world as moderate to strong sense of presence while practicing DBT mindfulness skills in VR. In addition to accepting the intervention, participants showed a significant increase in mindfulness state, and showed improved emotional state after their VR session. These findings are encouraging given the brief intervention used.

Furthermore, there are some interesting findings worthy to highlight regarding how changes in state of mindfulness and emotional state, as well as the sense of presence were related to the acceptability of the VR system to practice mindfulness. Despite the change in state of mindfulness was not significantly correlated with acceptability, there were significant correlations between change in emotional state (i.e., joy, surprise, relax, and vigor) and acceptability scores. Sense of presence was also significantly correlated with acceptability, which may point out the key role that both emotional state and sense of presence plays in the users’ acceptance using VR to practice mindfulness. Nevertheless, more studies are needed in order to explore how the VR approach is linked to mindfulness practice, and whether acceptability could be related to such relationship.

Although the findings are encouraging, this study has several important limitations. Acceptability/feasibility studies are scientifically inconclusive by nature [43], and the results should be interpreted with caution. The sample size (N = 44) was small, and the within-subject experimental design, and the absence of a control group limit conclusions.

Despite these limitations, the current results open a brand new line of research. Exploratory pilot studies are often an important first step toward later conducting full-scale trials [26]. Larger full scale randomized controlled trials are now warranted, and there are a number of interesting directions for future research. For example, several studies in the literature have found interesting differences in the effects of mindfulness for experts vs. novices [30, 44–46]. Future research exploring responsiveness to VR DBT mindfulness skills training for experts vs. non-expert meditators is needed. Future controlled studies could also help isolate how much the changes observed (e.g., reductions in negative emotions) are the result of the use of immersive virtual reality, exposure to the calm river scene, or the DBT® mindfulness skills training. Furthermore, testing different types of mindfulness exercises (using VR) to practice other important and more complex aspects of mindfulness, such us focusing on body sensations or mindfulness of current emotions, could be another future direction.

The current study explored the use of virtual reality mindfulness in non-clinical participants. In clinical studies (with no VR) DBT® skills training is proving to be a valuable element of Dialectical Behavioral Therapy for treating borderline personality disorder [11, 14, 15], and a growing number of other psychological problems for clinical (16) and non-clinical populations [47]. Traditional DBT® mindfulness skills training has also shown promising results with clinical populations [17–19]. VR mindfulness skills training may prove valuable for treating clinical patient populations. Many clinical populations have difficulty focusing their attention during mindfulness training (e.g., patients with borderline personality disorder) [18] and thus difficulty achieving mindful states, limiting clinical effectiveness. Because virtual reality is unusually attention grabbing, and blocks patients view of distracting information in the real world environment, VR DBT® mindfulness skills training may prove especially valuable for teaching mindfulness skills to clinical patients [27]. Mindfulness skills training using VR may enhance the effectiveness of the previously observed outcomes of traditional mindfulness training such as enhanced attention and improved emotional state.

VR technology is in the process of becoming widely available to mainstream consumers at an affordable price. Thus in addition to using VR to learn how to practice mindfulness, patients and non-patients may have their own VR system at home they can use to practice mindfulness. Currently the demand for DBT® far exceeds available resources. Computerized VR DBT® mindfulness skills training could increase dissemination of DBT® mindfulness skills training [27]. Further development and large scale randomized controlled studies exploring the use of VR DBT® mindfulness skills training are needed.

## Supporting information

S1 File Experimental data. (SAV)
